# Clifford Algebras Meet Tree Decompositions

**DOI:** 10.1007/s00453-018-0489-3

**Published:** 2018-07-30

**Authors:** Michał Włodarczyk

**Affiliations:** Faculty of Mathematics, Informatics, and Mechanics, University of Warsaw, Warsaw, Poland

## Abstract

We introduce the non-commutative subset convolution—a convolution of functions useful when working with determinant-based algorithms. In order to compute it efficiently, we take advantage of Clifford algebras, a generalization of quaternions used mainly in the quantum field theory. We apply this tool to speed up algorithms counting subgraphs parameterized by the treewidth of a graph. We present an $O^*((2^\omega + 1)^{tw})$-time algorithm for counting Steiner trees and an $O^*((2^\omega + 2)^{tw})$-time algorithm for counting Hamiltonian cycles, both of which improve the previously known upper bounds. These constitute also the best known running times of deterministic algorithms for decision versions of these problems and they match the best obtained running times for pathwidth parameterization under assumption $\omega = 2$.

## Introduction

The concept of *treewidth* has been introduced by Robertson and Seymour in their work on graph minors [13]. The treewidth of a graph is the smallest possible width of its *tree decomposition*, i.e., a tree-like representation of the graph. Its importance follows from the fact that many NP-hard graph problems become solvable on trees with a simple dynamical programming algorithm. A similar idea of *pathwidth* captures the width of a graph in case we would like to have a *path decomposition*. Formal definitions can be found in Sect. 2.2.

Having a tree decomposition of bounded width allows to design efficient algorithms using *fixed-parameter tractability*. An algorithm is called fixed-parameter tractable (FPT) if it works in time complexity $f(k)n^{O(1)}$ where *k* is a parameter describing hardness of the instance and *f* is a computable function. We also use notation $O^*(f(k))$ that suppresses polynomial factors with respect to the input size. Problems studied in this work are parameterized by the graph’s pathwidth or treewidth. To distinguish these cases we denote the parameter respectively *pw* or *tw*.

It is natural to look for a function *f* that is growing relatively slow. For problems with a local structure, like Vertex Cover or Dominating Set, there are simple FPT algorithms parameterized by the size of the solution with a single exponential running time. The basic idea is to store $c^{tw}$ states for each node of the decomposition and take advantage of the Fast Subset Convolution (FSC) algorithm [2] to perform the *join* operation in time $O^*(c^{tw})$. As a result, time complexities for pathwidth and treewidth parameterizations remain the same. The Fast Subset Convolution has proven useful in many other problems, e.g., Chromatic Number, and enriched the basic toolbox used for exponential and parameterized algorithms.

Problems with connectivity conditions, like Steiner Tree or Hamiltonian Cycle, were conjectured to require time $2^{\Omega (tw\log tw)}$ until the breakthrough work of Cygan et al. [8]. They introduced the randomized technique Cut & Count working in single exponential time. The obtained running times were respectively $O^*(3^{tw})$ and $O^*(4^{tw})$. Afterwards, a faster randomized algorithm for Hamiltonian Cycle parameterized by the pathwidth was presented with running time $O^*((2 + \sqrt{2})^{pw})$ [7]. This upper bound as well as $O^*(3^{pw})$ for Steiner Tree are tight modulo subexponential factors under the assumption of *Strong Exponential Time Hypothesis* [7, 8].

The question about the existence of single exponential deterministic methods was answered positively by Bodlaender et al. [4]. However, in contrast to the Cut & Count technique, a large gap has emerged between the running times for pathwidth and treewidth parameterizations—the running times were respectively $O^*(5^{pw})$, $O^*(10^{tw})$ for Steiner Tree and $O^*(6^{pw})$, $O^*(15^{tw})$ for Hamiltonian Cycle. This could be explained by a lack of efficient algorithms to perform the *join* operation, necessary only for tree decompositions. Some efforts have been made to reduce this gap and the deterministic running time for Steiner Tree has been improved to $O^*((2^{\omega - 1} \cdot 3 + 1)^{tw})$ [9].

The algorithms proposed in [4] also count the number of Steiner trees or Hamiltonian cycles in a graph by expressing the value in question as a sum of squared determinants of a particular family of submatrices of the graph incidence matrix. Very recently, Curticapean et al. [6] pointed out limitations of this technique even with respect to pathwidth parameterization. Namely, they proved that an algorithm counting Hamiltonian cycles in time $O^*((6-\epsilon )^{pw})$ would contradict the Strong Exponential Time Hypothesis.

### Our Contribution

The main contribution of this work is creating a link between Clifford algebras, objects not being used in algorithmics to the best of our knowledge, and fixed-parameter tractability. As the natural dynamic programming approach on tree decompositions uses the Fast Subset Convolution to perform efficiently the *join* operation, there was no such a tool for algorithms based on the determinant approach.

Our first observation is that the FSC technique can be regarded as an isomorphism theorem for some associative algebras. To put it briefly, a Fourier-like transform is being performed in the FSC to bring computations to a simpler algebra. Interestingly, this kind of transform is just a special case of the Artin–Wedderburn theorem [1], which seemingly is not widely reported in computer science articles. The theorem provides a classification of a large class of associative algebras, not necessarily commutative (more in “Appendix A”). We use this theory to introduce the Non-commutative Subset Convolution (NSC) and speed up multiplication operations in an algebra induced by the *join* operation in determinant-based dynamic programming on tree decomposition. An important building block is a fast Fourier-like transform for a closely related algebra [11]. We hope our work will encourage researchers to investigate further algorithmic applications of the Artin–Wedderburn theorem.

### Our Results

We apply our algebraic technique to the determinant approach introduced by Bodlaender et al. [4]. For path decomposition, they gave an $O^*(5^{pw})$-time algorithm for counting Steiner trees and an $O^*(6^{pw})$-time algorithm for counting Hamiltonian cycles. The running times for tree decomposition were respectively $O^*(10^{tw})$ and $O^*(15^{tw})$. These gaps can be explained by the appearance of the *join* operation in tree decompositions which could not be handled efficiently so far.

By performing NSC in time complexity $O^*(2^\frac{\omega n}{2})$ we partially solve an open problem stated by the FPT community.^1^ Our further results may be considered similar to those closing the gap between time complexities for pathwidth and treewidth parameterizations for Dominating Set by switching between representations of states in dynamic programming [14]. We improve the running times to $O^*((2^\omega + 1)^{tw})$ for counting Steiner trees and $O^*((2^\omega + 2)^{tw})$ for counting Hamiltonian cycles, where $\omega$ denotes the matrix multiplication exponent (currently it is established that $\omega < 2.373$ [15]). These are not only the fastest known algorithms for counting these objects, but also the fastest known deterministic algorithms for the decision versions of these problems.

Observe that the running times for pathwidth and treewidth parameterizations match under the assumption $\omega = 2$. Though we do not hope for settling the actual value of $\omega$, this indicates there is no further space for significant improvement unless pure combinatorial algorithms (i.e., not based on matrix multiplication) are invented or the running time for pathwidth parameterization is improved.

### Organization of the Paper

Section 3 provides a brief introduction to Clifford algebras. The bigger picture of the employed algebraic theory can be found in “Appendix A”. In Sect. 4 we define the NSC and design efficient algorithms for variants of the NSC employing the algebraic tools. Sections 5 and 6 present how to apply the NSC in counting algorithms for Steiner Tree and Hamiltonian Cycle. They contain main ideas improving the running times, however in order to understand the algorithms completely one should start from Sect. 4 (*Determinant approach*) in [4]. The algorithm for Hamiltonian Cycle is definitely more complex and its details, formulated as two isomorphism theorems, are placed in “Appendix C”.

## Preliminaries

We will start with notation conventions.

1. $$A \uplus B = C$$ stands for $$(A \cup B = C) \wedge (A \cap B = \emptyset )$$.
2. $$A \triangle B = (A \backslash B) \cup (B \backslash A)$$.
3. $[\alpha ]$ equals 1 if condition $\alpha$ holds and 0 otherwise.
4. For permutation *f* of a linearly ordered set *U*
  $$\begin{aligned} \text {sgn}(f) = (-1)^{|\{(a,b) \in U \times U \,\wedge \, a < b \,\wedge \, f(a) > f(b)\}|}. \end{aligned}$$
5. For *A*, *B* being subsets of a linearly ordered set
  1 $$\begin{aligned} I_{A,B} = (-1)^{|\{(a,b) \in A \times B \,\wedge \, a > b\}|}. \end{aligned}$$

Let us note two simple properties of *I*.

### Observation 1

For disjoint *A*, *B*

$$\begin{aligned} I_{A,B}I_{B,A} = (-1)^{|A||B|}. \end{aligned}$$

### Observation 2

For $$A\cap B = \emptyset $$ and $$C\cap D = \emptyset $$

$$\begin{aligned} I_{A\cup B, C\cup D} = I_{A,C}I_{A,D}I_{B,C}I_{B,D}. \end{aligned}$$

### Fast Subset Convolution

Let us consider a universe *U* of size *n* and functions $f,g:2^U \longrightarrow \mathbb {Z}$.

#### Definition 1

The Möbius transform of *f* is function $\hat{f}$ defined as

$$\begin{aligned} \hat{f}(X) = \sum _{A \subseteq X}f(A). \end{aligned}$$

#### Definition 2

Let $f * g$ denote a *subset convolution* of *f*, *g* defined as

$$\begin{aligned} (f * g)(X) = \sum _{A \uplus B = X}f(A)g(B). \end{aligned}$$

#### Theorem 3

(Björklund et al. [2]) The Möbius transform, its inverse, and the subset convolution can be computed in time $O^*(2^n)$.

### Pathwidth and Treewidth

#### Definition 3

A *tree (path) decomposition* of a graph *G* is a tree $\mathbb {T}$ (path $\mathbb {P}$) in which each node *x* is assigned a bag $B_x \subseteq V(G)$ such that

1. for every edge $uv \in E(G)$ there is a bag $B_x$ containing *u* and *v*,
2. for every vertex *v* the set $\{x\, |\, v \in B_x\}$ forms a non-empty subtree (subpath) in the decomposition.

The width of the decomposition is defined as $\max _x |B_x| - 1$ and the treewidth (pathwidth) of *G* is a minimum width over all possible tree (path) decompositions.

If a graph admits a tree decomposition of width *t* then it can be found in time $n\cdot 2^{O(t^3)}$ [3] and a decomposition of width at most $4t+1$ can be constructed in time $poly(n)\cdot 2^{O(t)}$ [10]. We will assume that a decomposition of the appropriate type and width is given as a part of the input.

#### Definition 4

A *nice tree (path) decomposition* is a decomposition with one special node *r* called the root and in which each bag is one of the following types:

1. *Leaf bag* a leaf *x* with $B_x = \emptyset$,
2. *Introduce vertexvbag* a node *x* having one child *y* for which $B_x = B_y \uplus \{v\}$,
3. *Forget vertexvbag* a node *x* having one child *y* for which $B_y = B_x \uplus \{v\}$,
4. *Introduce edgeuvbag* a node *x* having one child *y* for which $u,v \in B_x = B_y$,
5. *Join bag* (only in tree decomposition) a node *x* having two children *y*, *z* with condition $B_x = B_y = B_z$.

We require that every edge from *E*(*G*) is introduced exactly once and $B_r$ is an empty bag. For each *x* we define $V_x$ and $E_x$ to be sets of respectively vertices and edges introduced in the subtree of the decomposition rooted at *x*.

Given a tree (path) decomposition we can find a nice decomposition of the same width in time $n\cdot tw^{O(1)}$ [8, 10] and we will work only with these. When analyzing running time of algorithms working on tree decompositions we will estimate the bag sizes from the above assuming $|B_x| = tw$.

### Problems Definitions


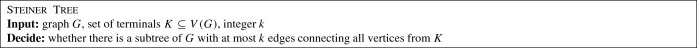
 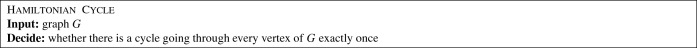


In the counting variants of problems we ask for a number of structures satisfying the given conditions. This setting is at least as hard as the decision variant.

## Clifford Algebras

Some terms used in this section originate from the advanced algebra. For better understanding we suggest reading “Appendix A”.

### Definition 5

The Clifford algebra $Cl_{p,q}(R)$ is a $2^{p+q}$-dimensional associative algebra over a ring *R*. It is generated by $x_1, x_2 \dots , x_{p+q}$.

These are rules of multiplication of generators:

1. *e* is a neutral element of multiplication,
2. $$x_i^2 = e$$ for $$i = 1, 2, \dots , p$$,
3. $$x_i^2 = -e$$ for $$i = p+1, p+2, \dots , p+q$$,
4. $$x_ix_j = -x_jx_i$$ if $$i \ne j$$.

All products (with respect to the ordering of elements) of $2^{p+q}$ sets of generators form a linear basis of $Cl_{p,q}(R)$ and *e* is treated as a product of an empty set. We provide a standard addition and we extend multiplication for all elements in an associative way.

We will be mostly interested in $Cl_{n,0}(\mathbb {Z})\,$^2^ and its natural embedding into $Cl_{n,0}(\mathbb {R})$. As $q=0$, we can neglect condition 3 when analyzing these algebras.

For $A = \{a_1, a_2, \dots , a_k\} \subseteq [1\dots n]$ where $a_1< a_2< \dots < a_k$ let $x_A = x_{a_1}x_{a_2}\dots x_{a_k}$. Each element of $Cl_{n,0}(\mathbb {R})\,$can be represented as $\sum _{A \subseteq [1\dots n]}a_Ax_A$, where $a_A$ are real coefficients. Using condition 4 we can deduce a general formula for multiplication in $Cl_{n,0}(\mathbb {R})\,$:

2 $$\begin{aligned} \left( \sum _{A \subseteq [1\dots n]}a_Ax_A\right) \left( \sum _{B \subseteq [1\dots n]}b_Bx_B\right) = \sum _{C \subseteq [1\dots n]}\left( \sum _{A\triangle B = C}a_Ab_BI_{A,B}\right) x_C \end{aligned}$$

where the meaning of $I_{A,B}$ is explained in (1).

Since the Clifford algebra over $\mathbb {R}$ is semisimple, it is isomorphic to a product of matrix algebras by the Artin–Wedderburn theorem (see Theorem 11). However, it is more convenient to first embed $Cl_{n,0}(\mathbb {R})\,$in a different Clifford algebra that is isomorphic to a single matrix algebra. As a result, we obtain a monomorphism $\phi : Cl_{n,0}(\mathbb {R}) \longrightarrow \mathbb {M}_{2^m}(\mathbb {R})$ (see Definition 10) where $m = \frac{n}{2} + O(1)$ and the following diagram commutes ($*$ stands for multiplication).3Thus, we can perform multiplication in the structure that is more convenient for us. For $a,b \in Cl_{n,0}(\mathbb {Z})$ we can treat them as elements of $Cl_{n,0}(\mathbb {R})$, find matrices $\phi (a)$ and $\phi (b)$, multiply them efficiently, and then invert the $\phi$ transform. The result always exists and belongs to $Cl_{n,0}(\mathbb {Z})\,$because $Cl_{n,0}(\mathbb {Z})\,$is closed under multiplication. The monomorphism $\phi : Cl_{n,0}(\mathbb {R}) \longrightarrow \mathbb {M}_{2^m}(\mathbb {R})$ can be performed and inverted (within the image) in $O^*(2^n)$ time [11]. However, the algorithm in [11] is analyzed in the infinite precision model. For the sake of completeness, we revisit this construction and prove the following theorem in “Appendix B”.

### Theorem 4

The multiplication in $Cl_{n,0}(\mathbb {Z})$, with coefficients having *poly*(*n*) number of bits, can be performed in time $O^*(2^{\frac{\omega n}{2}})$.

In order to unify the notation we will represent each element of $Cl_{n,0}(\mathbb {Z})\,$, that is $\sum _{A \subseteq [1\dots n]}a_Ax_A$, as a function $f:2^{[1\dots n]} \longrightarrow \mathbb {Z},\, f(A) = a_A$. We introduce $\diamond _S$ convolution as an equivalence of multiplication in $Cl_{n,0}(\mathbb {Z})\,$. The equation (2) can be now rewritten in a more compact form

4 $$\begin{aligned} (f \diamond _S g)(X) = \sum _{A \triangle B = X}f(A)g(B)I_{A,B}. \end{aligned}$$

## Non-commutative Subset Convolution

We consider a linearly ordered universe *U* of size *n* and functions $f,g:2^U \longrightarrow \mathbb {Z}$.

### Definition 6

Let $f \diamond g$ denote *Non-commutative Subset Convolution* (NSC) of functions *f*, *g* defined as

$$\begin{aligned} (f \diamond g)(X) = \sum _{A \uplus B = X}f(A)g(B)I_{A,B}. \end{aligned}$$

### Theorem 5

NSC on an *n*-element universe can be performed in time $O^*(2^{\frac{\omega n}{2}})$.

### Proof

Observe that condition $A \uplus B = X$ is equivalent to $A \triangle B = X\, \wedge \, |A| + |B| = |X|$ so

$$\begin{aligned} (f \diamond g)(X) = \sum _{\begin{array}{c} i + j = |X| \\ i, j \ge 0 \end{array}} \sum _{A \triangle B = X}f(A)\Big [|A| = i\Big ]g(B)\Big [|B| = j\Big ]I_{A,B}. \end{aligned}$$

Alternatively, we can write

$$\begin{aligned} (f \diamond g)(X) = \sum _{\begin{array}{c} i + j = |X| \\ i, j \ge 0 \end{array}} (f_i \diamond _S g_j)(X), \end{aligned}$$

where $f_i(X) = f(X)\Big [|X| = i\Big ]$ and likewise for *g*. The $\diamond _S$ convolution, introduced in (4), is equivalent to multiplication in $Cl_{n,0}(\mathbb {R})\,$. This means we reduced NSC to $O(n^2)$ multiplications in $Cl_{n,0}(\mathbb {R})\,$which could be performed in time $O^*(2^{\frac{\omega n}{2}})$ according to Theorem 4. $\square$

### Remark 1

The technique of grouping the sizes of sets with polynomial burden in the running time will turn useful in further proofs. We will call it size-grouping.

In our applications we will need to compute a slightly more complex convolution.

### Definition 7

When *f*, *g* are of type $2^U \times 2^U \longrightarrow \mathbb {Z}$ we can define $f \diamond _2 g$ (NSC2) as follows

$$\begin{aligned} (f \diamond _2 g)(X, Y) = \sum _{\begin{array}{c} X_1 \uplus X_2 = X \\ Y_1 \uplus Y_2 = Y \end{array}} f(X_1,Y_1)g(X_2,Y_2)I_{X_1,X_2}I_{Y_1,Y_2}. \end{aligned}$$

### Theorem 6

NSC2 on an *n*-element universe can be performed in time $O^*(2^{\omega n})$.

### Proof

Let us introduce a new universe $U' = U_X \cup U_Y$ of size 2*n* consisting of two copies of *U* with an order so each element of $U_Y$ is greater than any element of $U_X$. To underline that $X \subseteq U_X, Y \subseteq U_Y$ we will use $\uplus$ notation when summing subsets of $U_X$ and $U_Y$. In order to reduce NSC2 to NSC on the universe $U'$ we need to replace factor $I_{X_1,X_2}I_{Y_1,Y_2}$ with $I_{X_1 \uplus Y_1,X_2 \uplus Y_2}$. The latter term can be expressed as $I_{X_1,X_2}I_{Y_1,Y_2}I_{X_1,Y_2}I_{Y_1,X_2}$ due to Observation 2. As all elements from $X_i \subseteq U_X$ compare less to elements from $Y_i \subseteq U_Y$ then $I_{X_1,Y_2} = 1$ and $I_{Y_1,X_2}$ depends only on the sizes of $Y_1$ and $X_2$. To summarize,

$$\begin{aligned} I_{X_1,X_2}I_{Y_1,Y_2} = I_{X_1 \uplus Y_1,X_2 \uplus Y_2}(-1)^{|Y_1||X_2|}. \end{aligned}$$

To deal with factor $(-1)^{|Y_1||X_2|}$ we have to split the convolution into 4 parts for different parities of $|Y_1|$ and $|X_2|$. We define functions $f', f'_0, f'_1, g', g'_0, g'_1 : 2^{U'} \longrightarrow \mathbb {Z}$ as

$$\begin{aligned} f'(X \uplus Y)= & {} f(X, Y),\\ f'_0(X \uplus Y)= & {} f(X, Y)\Big [\,|Y| \equiv 0 \bmod 2\Big ],\\ f'_1(X \uplus Y)= & {} f(X, Y)\Big [\,|Y| \equiv 1 \bmod 2\Big ],\\ g'(X \uplus Y)= & {} g(X, Y),\\ g'_0(X \uplus Y)= & {} g(X, Y)\Big [\,|X| \equiv 0 \bmod 2\Big ],\\ g'_1(X \uplus Y)= & {} g(X, Y)\Big [\,|X| \equiv 1 \bmod 2\Big ]. \end{aligned}$$

Now we can reduce NSC2 to 4 simpler convolutions.

$$\begin{aligned}&(f \diamond _2 g)(X, Y) = \sum _{\begin{array}{c} X_1 \uplus X_2 = X \\ Y_1 \uplus Y_2 = Y \end{array}} f'(X_1 \uplus Y_1)g'(X_2 \uplus Y_2)I_{X_1 \uplus Y_1, Y_2 \uplus X_2}(-1)^{|Y_1||X_2|} = \\&\quad = (f'_0 \diamond g'_0)(X \uplus Y) + (f'_0 \diamond g'_1)(X \uplus Y) + (f'_1 \diamond g'_0)(X \uplus Y) - (f'_1 \diamond g'_1)(X \uplus Y) \end{aligned}$$

We have shown that computing NSC2 is as easy as NSC on a universe of size twice the original universe size. Using Theorem 5 directly gives us the desired complexity. $\square$

## Counting Steiner Trees

We will revisit the theorem stated in the aforementioned work.

### Theorem 7

(Bodlaender et al. [4]) There exist algorithms that given a graph *G* count the number of Steiner trees of size *i* for each $1 \le i \le n - 1$ in $O^*(5^{pw})$ time if a path decomposition of width *pw* is given, and in $O^*(10^{tw})$ time if a tree decomposition of width *tw* is given.

Both algorithms use dynamic programming over tree or path decompositions. We consider vertices in a particular decomposition-based order and fix vertex $v_1 \in K$, where *K* is the set of terminals. Let $A = (a_{v,e})_{v \in V, e \in E}$ be the incidence matrix, i.e., for $e = uv,\, u < v$ we have $a_{u,e}=1,\, a_{v,e}=-1$, and $a_{w,e} = 0$ for any other vertex *w*. For $K\subseteq Y$ let $A_{Y,X}$ be a submatrix of *A* with rows in $Y \setminus \{v_1\}$ and columns in *X*. The value of $|\det (A_{Y,X})|$ turns out to be 1 if the subgraph *G*(*X*, *Y*) forms a tree and 0 otherwise. The main idea of the algorithm is to express the number of Steiner trees for terminal set *K* with exactly *i* edges as

$$\begin{aligned} \sum _{K \subseteq Y \subseteq V,\, |Y| = i-1,} \sum _{E \subseteq E(Y,Y),\, |X| = |Y|-1} \det (A_{Y,X})^2. \end{aligned}$$

We consider partial sums representing summands belonging to the subtree of a decomposition node *x*. We exploit the permutation formula for determinant and introduce functions $s_1, s_2$ to control the image of permutations (there are two permutations in each summand since we expand a square of determinant) within the bag $B_x$. We also introduce function $s_Y$ indicating which vertices belong to $Y \cap B_x$. For node *x* of the decomposition we define function $A_x$ with arguments $0 \le i \le n - 1,\, s_Y, s_1, s_2 \in \{0, 1\}^{B_x}$ as

5 $$\begin{aligned}&\sum _{\begin{array}{c} Y \subseteq V_x \\ |Y|=i \\ (K \cap V_x)\subseteq Y \\ Y \cap B_x = s_Y^{-1}(1) \end{array}} \sum _{X\subseteq E(Y,Y)\cap E_x} \sum _{\begin{array}{c} f_1:X \overset{1-1}{\rightarrow }Y\backslash \{v_1\}\backslash s_1^{-1}(0) \\ f_2:X \overset{1-1}{\rightarrow }Y\backslash \{v_1\}\backslash s_2^{-1}(0) \end{array}} \text {sgn}(f_1)\text {sgn}(f_2)\prod _{e\in X}a_{f_1(e),e}a_{f_2(e),e} \end{aligned}$$

Then the number of Steiner trees with exactly *i* edges becomes $A_r(i+1,\emptyset ,\emptyset ,\emptyset )$ [4]. As observed therein, condition $s_Y(v)=0$ implies that either $s_1(v) = s_2(v) = 0$ or $A_x(i, s_Y, s_1, s_2) = 0$. This means there are at most $n\cdot 5^{tw}$ triples for which $A_x$ returns a nonzero value.

For a node *x* of type *introduce vertex*, *introduce edge*, or *forget vertex*, with a child *y*, the function $A_x$ can be computed from $A_y$ in linear time with respect to the number of non-trivial states. This observation leads to a proof of Theorem 7 for path decompositions. The only thing that is more difficult for tree decompositions is that they include also *join* nodes having two children each. Here is the recursive formula^3^ for $A_x$ for a *join* node *x* with children *y*, *z*.

6 $$\begin{aligned} A_x(i, s_Y, s_1, s_2) = \sum _{\begin{array}{c} i_y + i_z = i + |s_Y^{-1}(1)| \\ s_{1,y} + s_{1,z} = s_1 \\ s_{2,y} + s_{2,z} = s_2 \end{array}} \genfrac{}{}{0.0pt}{}{A_y(i_y, s_Y, s_{1,y}, s_{2,y})A_z(i_z, s_Y, s_{1,z}, s_{2,z})}{I_{s_{1,y}^{-1}(1), s_{1,z}^{-1}(1)}I_{s_{2,y}^{-1}(1), s_{2,z}^{-1}(1)}} \end{aligned}$$

The next lemma, however not stated explicitly in the discussed work, follows from the proof of Theorem 7 (Theorem 4.4 in [4]).

### Lemma 1

Assume there is an algorithm computing all nonzero values of $A_x$ given by (6) with running time *f*(*tw*). Then the number of Steiner trees of size *i* in a graph *G* can be counted in $O^*(\max (f(tw), 5^{tw}))$ time if a tree decomposition of width *tw* is given.

We will change notation for our convenience. Each function $s_i$ will be matched with set $s_i^{-1}(1)$. Let us replace functions $A_x, A_y, A_z$ with $h_i,f_i,g_i$ having first argument fixed and operating on triples of sets. In this setting, the convolution can we written as

7 $$\begin{aligned} h_i(A,B,C) = \sum _{\begin{array}{c} i_y + i_z = i + |A| \\ B_y \uplus B_z = B \\ C_y \uplus C_z = C \end{array}} f_{i_y}(A,B_y,C_y)g_{i_z}(A,B_z,C_z)I_{B_y,B_z}I_{C_y,C_z}. \end{aligned}$$

Observe that size-grouping allows us to neglect the restrictions for $i,i_y,i_z$. Hence, we can work with a simpler formula

8 $$\begin{aligned} h(A,B,C) = \sum _{\begin{array}{c} B_y \uplus B_z = B \\ C_y \uplus C_z = C \end{array}} f(A,B_y,C_y)g(A,B_z,C_z)I_{B_y,B_z}I_{C_y,C_z}. \end{aligned}$$

The only triples $\left( s_Y(v), s_1(v), s_2(v)\right)$ allowed for each vertex *v* are (0, 0, 0), (1, 0, 0), (1, 0, 1), (1, 1, 0), (1, 1, 1). In terms of set notation we can say that if $f(A,B,C) \ne 0$ then $B \cup C \subseteq A$. Let $f_A : 2^A \times 2^A \longrightarrow \mathbb {Z}$ be *f* with the first set fixed, i.e., $f_A(B,C) = f(A,B,C)$.

### Lemma 2

For fixed *A* all values *h*(*A*, *B*, *C*) can be computed in time $O^*(2^{\omega |A|})$.

### Proof

We want to compute

$$\begin{aligned} h_A(B,C) = \sum _{\begin{array}{c} B_y \uplus B_z = B \\ C_y \uplus C_z = C \end{array}} f_A(B_y,C_y)g_A(B_z,C_z)I_{B_y,B_z}I_{C_y,C_z} = (f_A \diamond _2 g_A)(B, C), \end{aligned}$$

what can be done in time $O^*(2^{\omega |A|})$ according to Theorem 6. $\square$

### Lemma 3

The convolution (7) can be performed in time $O^*((2^{\omega } + 1)^{tw})$.

### Proof

We use size-grouping to reduce the problem to computing (8). Then we iterate through all possible sets *A* and take advantage of Lemma 2. The total number of operations (modulo polynomial factor) is bounded by

$$\begin{aligned} \sum _{A \subseteq U} 2^{\omega |A|} = \sum _{k=0}^{tw} \left( {\begin{array}{c}tw\\ k\end{array}}\right) 2^{\omega k} = (2^{\omega } + 1)^{tw}. \end{aligned}$$

$$\square $$

Keeping in mind that (6) and (7) are equivalent and combining Lemmas 1 and 3, we obtain the following result.

### Theorem 8

The number of Steiner trees of size *i* in a graph *G* can be computed in $O^*((2^{\omega } + 1)^{tw})$ time if a tree decomposition of width *tw* is given.

## Counting Hamiltonian Cycles

Likewise in the previous section, we will start with a previously known theorem.

### Theorem 9

(Bodlaender et al. [4]) There exist algorithms that given a graph *G* count the number of Hamiltonian cycles in $O^*(6^{pw})$ time if a path decomposition of width *pw* is given, and in $O^*(15^{tw})$ time if a tree decomposition of width *tw* is given.

We again consider vertices in a particular decomposition-based order and fix an arbitrary vertex $v_1$. For a subset $S \subseteq E$ let $A_S$ be the submatrix of the incidence matrix *A* (see previous section) with rows from $V \setminus \{v_1\}$ and columns from *S*. We express the number of Hamiltonian cycles as

$$\begin{aligned} \frac{1}{n} \sum _{\genfrac{}{}{0.0pt}{}{X \subseteq E}{\text {s.t. } \forall _{v \in V} deg_X(v) = 2}} \sum _{S \subseteq X,\, |S|=n-1} \det (A_S)^2. \end{aligned}$$

We again consider partial sums representing summands belonging to the subtree of a decomposition node *x* and introduce functions $s_1,s_2$ to keep track of permutations’ images within the bag $B_x$. The function $s_{deg}$ controls the degree of a vertex within $B_X$. The notation is analogous to (5). For each node *x* of the decomposition function $A_x$ is defined with arguments $s_1, s_2 \in \{0,1\}^{B_x}$ and $s_{deg} \in \{0,1,2\}^{B_x}$ as

9 $$\begin{aligned}&\sum _{\begin{array}{c} X \subseteq E_x \\ \forall _{v \in (V_x\backslash B_x)} deg_X(v)=2 \\ \forall _{v\in B_x} deg_X(v)=s_{deg}(v) \end{array}} \sum _{S\subseteq X} \sum _{\begin{array}{c} f_1:S \overset{1-1}{\rightarrow }V_x\backslash \{v_1\}\backslash s_1^{-1}(0) \\ f_2:S \overset{1-1}{\rightarrow }V_x\backslash \{v_1\}\backslash s_2^{-1}(0) \end{array}} \text {sgn}(f_1)\text {sgn}(f_2)\prod _{e\in S}a_{f_1(e),e}a_{f_2(e),e} \end{aligned}$$

The number of Hamiltonian cycles can be then expressed as $A_r(\emptyset ,\emptyset ,\emptyset ) / n$.

As observed in [4] we can restrict ourselves only to some subspace of states. When $s_{deg}(v)=0$ then all non-zero summands in (9) satisfy $s_1(v) = s_2(v) = 0$. When $s_{deg}(v)=2$ then we can neglect all summands except for those satisfying $s_1(v) = s_2(v) = 1$.

This time there are at most $6^{tw}$ triples for which $A_x$ returns a nonzero value. We again argue that *introduce vertex*, *introduce edge*, and *forget vertex* nodes can be handled the same way as for the path decomposition and the only bottleneck is formed by *join* nodes. We present a formula for $A_x$ if *x* is a *join* node with children *y*, *z*.

10 $$\begin{aligned} A_x(s_{deg}, s_1, s_2) = \sum _{\begin{array}{c} s_{deg,y} + s_{deg,z} = s_{deg} \\ s_{1,y} + s_{1,z} = s_1 \\ s_{2,y} + s_{2,z} = s_2 \end{array}} \genfrac{}{}{0.0pt}{}{ A_y(s_{deg,y}, s_{1,y}, s_{2,y})A_z(s_{deg,z}, s_{1,z}, s_{2,z})}{I_{s_{1,y}^{-1}(1), s_{1,z}^{-1}(1)}I_{s_{2,y}^{-1}(1), s_{2,z}^{-1}(1)}} \end{aligned}$$

Analogously to the algorithm for Steiner Tree, we formulate our claim as a lemma following from the proof of Theorem 9 (Theorem 4.3 in [4]).

### Lemma 4

Assume there is an algorithm computing all nonzero values of $A_x$ given by (10) with running time *f*(*tw*). Then the number of Hamiltonian cycles in a graph *G* can be counted in $O^*(\max (f(tw), 6^{tw}))$ time if a tree decomposition of width *tw* is given.

The only allowed triples of $\left( s_{deg}(v), s_1(v), s_2(v)\right)$ for each vertex *v* are (0, 0, 0),  (1, 0, 0),  (1, 0, 1),  (1, 1, 0),  (1, 1, 1),  (2, 1, 1).

### Lemma 5

Assume the Eq. (10) holds. Then it remains true after the following translation of the set of allowed triples $\left( s_{deg}(v), s_1(v), s_2(v)\right)$.

$$\begin{aligned} 0,0,0 \longrightarrow 0,0,0 \\ 1,0,0 \longrightarrow 1,0,0 \\ 1,0,1 \longrightarrow 1,0,1 \\ 1,1,0 \longrightarrow 0,1,0 \\ 1,1,1 \longrightarrow 0,1,1 \\ 2,1,1 \longrightarrow 1,1,1 \end{aligned}$$

### Proof

The $I_{.,.}$ factors do not change as we do not modify the coordinates given by functions $s_1, s_2$. Triples that match in (10) translate into matching triples as the transformation keeps their additive structure. This fact can be illustrated by rewriting the addition table from [4] with the new coordinates. $\square$

**Table:** 

	000	100	101	110	111	211
000	000	100	101	110	111	211
100	100	X	X	X	211	X
101	101	X	X	211	X	X
110	110	X	211	X	X	X
111	111	211	X	X	X	X
211	211	X	X	X	X	X

**Table:** 

	000	100	101	010	011	111
000	000	100	101	010	011	111
100	100	X	X	X	111	X
101	101	X	X	111	X	X
010	010	X	111	X	X	X
011	011	111	X	X	X	X
111	111	X	X	X	X	X

Therefore we can treat function $s_{deg}$ as a binary one. We unify the notation by representing functions $s_i$ with the corresponding sets $s_i^{-1}(1)$. We replace functions $A_x, A_y, A_z$ with their counterparts *h*, *f*, *g* operating on triples of sets. For example, expression $A_x(s_{deg},s_1,s_2)$ gets transformed into $h(s_{deg}^{-1}(1), s_1^{-1}(1), s_2^{-1}(1))$. In this setting, the convolution looks as follows.

11 $$\begin{aligned} h(A,B,C) = \sum _{\begin{array}{c} A_1 \uplus A_2 = A \\ B_1 \uplus B_2 = B \\ C_1 \uplus C_2 = C \end{array}} f(A_1,B_1,C_1)g(A_2,B_2,C_2)I_{B_1,B_2}I_{C_1,C_2} \end{aligned}$$

Performing convolution (11) within the space of allowed triples involves more sophisticated techniques than those in Sect. 5. Therefore the proof of the following lemma is postponed to “Appendix C”.

### Lemma 6

The convolution (11) can be computed in time $O^*((2^{\omega } + 2)^{tw})$.

This result, together with Lemmas 4 and 5, leads to the main theorem of this section.

### Theorem 10

The number of Hamiltonian cycles in a graph *G* can be computed in $O^*((2^{\omega } + 2)^{tw})$ time if a tree decomposition of width *tw* is given.

## Conclusions

We have presented the Non-commutative Subset Convolution, a new algebraic tool in algorithmics based on the theory of Clifford algebras. This allowed us to construct faster deterministic algorithms for Steiner Tree, Feedback Vertex Set, and Hamiltonian Cycle, parameterized by the treewidth. As the determinant-based approach applies to all problems solvable by the Cut & Count technique [4, 8], the NSC can improve running times for a larger class of problems.

The first open question is whether the gap between time complexities for the decision and counting versions of these problems could be closed. Or maybe one can prove this gap inevitable under a well-established assumption, e.g., SETH?

The second question is if it is possible to prove a generic theorem so the lemmas like 3 or 6 would follow from it easily. It might be possible to characterize convolution algebras that are semisimple and algorithmically construct isomorphisms with their canonical forms described by the Artin–Wedderburn theorem.

The last question is what other applications of Clifford algebras and Artin–Wedderburn theorem can be found in algorithmics.

## Associative Algebras

This section is not crucial to understanding the paper but it provides a bigger picture of the applied theory. We assume that readers are familiar with basic algebraic structures like rings or fields. More detailed introduction can be found, e.g., in [1].

### Definition 8

A linear space *A* over a field *K* (or, more generally, a module over a ring *K*) is called an *associative algebra* if it admits a multiplication operator $A \times A \rightarrow A$ satisfying the following conditions:

1. $$\forall _{a,b,c \in A}\,\, a(bc)=(ab)c$$,
2. $$\forall _{a,b,c \in A}\,\, a(b+c)=ab+ac,\, (b+c)a = ba + ca$$,
3. $$\forall _{a,b \in A, k \in K}\,\, k(ab) = (ka)b = a(kb)$$.

A set $W \subseteq A$ is called a *generating set* if every element of *A* can be obtained from *W* by addition and multiplication. The elements of *W* are called *generators*. It is easy to see that multiplication defined on a generating set extends in an unambiguous way to the whole algebra. We will often abbreviate the term *associative* as we will study only such algebras.

### Definition 9

The product of algebras $A_1,A_2,\dots ,A_m$ is an algebra $A_1 \otimes A_2 \otimes \dots \otimes A_m$ with multiplication performed independently on each coordinate.

### Definition 10

For algebras *A*, *B* over a ring *K*, function $\phi :A \rightarrow B$ is called a $\textit{homomorphism of algebras}$ if it satisfy the following conditions:

1. $$\forall _{a,b \in A}\,\, \phi (a+b) = \phi (a) + \phi (b)$$,
2. $$\forall _{a,b \in A}\,\, \phi (ab) = \phi (a)\phi (b)$$,
3. $$\forall _{a\in A, k \in K}\,\, \phi (ka) = k\phi (a)$$.

If $\phi$ is reversible within its image then we call it a $\textit{monomorphism}$ and if additionally $\phi (A) = B$ then we call $\phi$ an $\textit{isomorphism}$

Monomorphisms of algebras turn out extremely useful when multiplication in algebra *B* is simpler than multiplication in *A*, because we can compute *ab* as $\phi ^{-1}\big (\phi (a)\phi (b)\big )$. This observation is used in Theorem 4 and Lemmas 6, 7. For a better intuition, we depict the various ways of performing multiplication on diagrams (3, 14).

### Definition 11

A subset *M* of algebra *A* is called a *simple left module* if

1. $$\forall _{a \in A, b \in M}\,\, ab \in M$$,
2. $$\forall _{b,c \in M}\,\, b+c \in M$$,

and the only proper subset of *M* with these properties is $\{0\}$.

The next definition is necessary to exclude some cases of obscure algebras.

### Definition 12

An algebra *A* is called *semisimple* if there is no non-zero element *a* so for every simple left module $M \subseteq A$ the set $aM = \{ab\, |\, b \in M\}$ is $\{0\}$.

The theorem below has been stated in full generality for algebras over arbitrary rings but we will formulate its simpler version for fields.

### Theorem 11

(Artin–Wedderburn [1]) Every finite-dimensional associative semisimple algebra *A* over a field *K* is isomorphic to a product of matrix algebras

$$\begin{aligned} A \cong M_{n_1}(K_1) \otimes M_{n_2}(K_2) \otimes \dots \otimes M_{n_m}(K_m), \end{aligned}$$

where $K_i$ are fields containing *K*.

The related isomorphism is called a generalized Fourier transform (GFT) for *A*. If we are able to perform GFT efficiently then we can reduce computations in *A* to matrix multiplication. For some classes of algebras, e.g., abelian group algebras, there are known algorithms for GFT with running time $O(n\log n)$ where $n = \dim A$ [12].

If the field *K* is algebraically closed (e.g., $\mathbb {C}$) then all $K_i = K$ and $\sum _{i=1}^m n_i^2$ equals the dimension of *A*. If the algebra *A* is commutative then all $n_i = 1$ and *A* is isomorphic to a product of fields. This is actually the case in the Fast Subset Convolution [2], where the isomorphism is given by the Möbius transform.

## Proof of Theorem 4

### Proof

The transformation $\phi$ can be computed and inverted (within the image) in time $O^*(2^n)$ assuming infinite precision and *O*(1) time for any arithmetic operation [11]. In order to compute $\phi$ accurately in the RAM model, we need to look inside the paper [11]. The formulas therein are based on the work of Cnops [5].

Transformation $\phi$ can be represented as $\phi = \gamma \circ \upsilon$ where $\upsilon$ is a monomorphic embedding into another Clifford algebra and $\gamma$ is an isomorphism with the matrix algebra. We modify the commutative isomorphism diagram (3) to illustrate these mappings in more detail.

$$\begin{aligned} \begin{array}{ccccccc} Cl_{n,0}(\mathbb {Z}) &{}\hookrightarrow &{} Cl_{n,0}(\mathbb {R}) &{}\overset{\upsilon }{\longrightarrow } &{}Cl_{m,m}(\mathbb {R}) &{}\overset{\gamma }{\longrightarrow } &{}\mathbb {M}_{2^m}(\mathbb {R}) \\ \downarrow * &{}&{}\downarrow * &{}&{}\downarrow * &{}&{}\downarrow *\\ Cl_{n,0}(\mathbb {Z}) &{}\hookrightarrow &{} Cl_{n,0}(\mathbb {R}) &{}\overset{\upsilon }{\longrightarrow } &{}Cl_{m,m}(\mathbb {R}) &{}\overset{\gamma }{\longrightarrow } &{}\mathbb {M}_{2^m}(\mathbb {R}) \end{array} \end{aligned}$$

We begin with embedding $\upsilon : Cl_{n,0}(\mathbb {R}) \longrightarrow Cl_{m,m}(\mathbb {R})$ where $m = \frac{n}{2} + O(1)$ (see Definition 4.4 in [11]). Transformation $\upsilon$ is just a translation of basis so no arithmetic operations are involved.

For the sake of disambiguation, we indicate the domain of the function $\gamma$ with a lower index: $\gamma _k: Cl_{k,k}(\mathbb {R}) \longrightarrow \mathbb {M}_{2^k}(\mathbb {R})$. In the *k*-th step, we construct a matrix representation of $y \in Cl_{k,k}(\mathbb {R})$. Let $y^+, y^-$ denote the projections of *y* onto subspaces spanned by products of respectively even and odd number of generators.^4^ Of course, $y = y^+ + y^-$ and $\gamma _k(y) = \gamma _k(y^+) + \gamma _k(y^-)$, since the mapping is linear. Such an element *y* can be represented as $y = a + b\mathbf {x_-} + c\mathbf {x_+} + d\mathbf {x_-}\mathbf {x_+}$ for $\mathbf {x_+},\mathbf {x_-}$ being the generators with indices 1,k (we have $\mathbf {x}_+^2 = e,\, \mathbf {x}_-^2 = -e$) and $a,b,c,d \in Cl_{k-1,k-1}(\mathbb {R})$. Now we can apply the recursive formula from Theorem 5.2 in [11]:

$$\begin{aligned}&\gamma _k(y^+) = \gamma _{k-1}\left( \left[ \begin{array}{cc} a^+ - d^+,\quad &{} -b^- - c^- \\ -b^- - c^-,\quad &{} a^+ + d^+ \end{array} \right] \right) , \\&\\&\gamma _k(y^-) = \gamma _{k-1}\left( \left[ \begin{array}{cc} a^- - d^-,\quad &{} -b^+ + c^+ \\ b^+ + c^+,\quad &{} -a^- - d^- \end{array} \right] \right) , \end{aligned}$$

where $\gamma _{k-1}(M)$ stands for a block matrix with $\gamma _{k-1}$ applied to each element of *M*.

We see that computing $\big (\gamma _k(y^+), \gamma _k(y^-)\big )$ can be reduced to computing 4 analogous pairs for $k-1$ and combining them using addition and subtraction. Hence, the coefficients of the obtained matrix will also be integers with *poly*(*n*) number of bits and the total number of arithmetic operations is $O(m4^m) = O(n2^n)$.

The inverse transform $\gamma ^{-1}$ is also computed in *m* steps and we continue using lower index to indicate the domain alike for the forward transform. Let $Y \in \mathbb {M}_{2^k}(\mathbb {Z})$ and

$$\begin{aligned} Y = \left[ \begin{array}{cc} Y_{11} &{} Y_{12} \\ Y_{21} &{} Y_{22} \end{array} \right] ,\quad y_{ij} = \gamma _{k-1}^{-1}(Y_{ij}). \end{aligned}$$

Then from Theorem 7.1 in [11] we know that

$$\begin{aligned} \gamma _{k}^{-1}(Y) = \frac{1}{2}\big ((\hat{y_{22}} + y_{11}) + (\hat{y_{21}} - y_{12})\mathbf {x_-} + (\hat{y_{21}} + y_{12})\mathbf {x_+} + (\hat{y_{22}} - y_{11})\mathbf {x_-}\mathbf {x_+}\big ), \end{aligned}$$

where $\hat{y} = y^+ - y^-$ and the rest of notation is as above. We can reduce computing $\gamma _{k}^{-1}$ to 4 queries from $(k-1)$-th step so the total number of arithmetic operations is $O(m4^m) = O(n2^n)$.

This time the coefficients at each step are given as sums of elements from the previous step divided by 2. We do not need to prove that they remain integer at all steps because we can postpone the division until the last step. As long as $\gamma ^{-1}(Y)$ is a product of two elements from $Cl_{m,m}(\mathbb {Z})$, it is guaranteed that the numbers in the last step are integers and our variables are divisible by $2^m$. What is more, if we know that $\gamma ^{-1}(Y) \in \upsilon (Cl_{n,0}(\mathbb {Z}))$ then we can revert the $\upsilon$ transform and obtain $\phi ^{-1}(Y)$.

We have proven that we can switch representation between $Cl_{n,0}(\mathbb {Z})$ and $\mathbb {M}_{2^m}(\mathbb {Z})$ in time $O^*(2^n)$. The multiplication in $\mathbb {M}_{2^m}(\mathbb {Z})$ for inputs of *poly*(*n*) size can be performed in time complexity $O^*(2^{\omega m}) = O^*(2^{\frac{\omega n}{2}})$ and the resulting matrix also contains only *poly*(*n*)-bits integers. This proves that the multiplication in $Cl_{n,0}(\mathbb {Z})$ admits an algorithm with running time $O^*(2^{\frac{\omega n}{2}})$. $\square$

## Proof of Lemma 6

This section reduces the algorithm for Hamiltonian Cycle to two isomorphism theorems and we suggest reading “Appendix A” first. Our goal is to compute values of *h* for the allowed triples assuming that non-zero values of *f*, *g* also occur only for the allowed triples.

12 $$\begin{aligned} h(A,B,C) = \sum _{\begin{array}{c} A_1 \uplus A_2 = A \\ B_1 \uplus B_2 = B \\ C_1 \uplus C_2 = C \end{array}} f(A_1,B_1,C_1)g(A_2,B_2,C_2)I_{B_1,B_2}I_{C_1,C_2} \end{aligned}$$

Taking advantage of the size-grouping technique (see Observation 1) we can replace condition $A_1 \uplus A_2 = A$ with $A_1 \cup A_2 = A$ and focus on the following convolution.

13 $$\begin{aligned} (f \odot g)(A,B,C) = \sum _{\begin{array}{c} A_1 \cup A_2 = A \\ B_1 \uplus B_2 = B \\ C_1 \uplus C_2 = C \end{array}} f(A_1,B_1,C_1)g(A_2,B_2,C_2)I_{B_1,B_2}I_{C_1,C_2} \end{aligned}$$

Let *Ham* be a subspace of $2^U \times 2^U \times 2^U \longrightarrow \mathbb {Z}$ given by functions admitting only the allowed triples (see Lemma 5), i.e., $f \in Ham \wedge f(A,B,C)\ne 0$ implies $A \cap (B \triangle C) = C \backslash B$. Observe that *Ham* is closed under the $\odot$ operation so it can be regarded as a $6^{tw}$-dimensional algebra. Let $H_D$ be an algebra over space $2^{U\backslash D} \times 2^D \times 2^D \longrightarrow \mathbb {Z}$ with multiplication given by the $\oslash$ operator defined as

$$\begin{aligned} (f \oslash g)(E,B,C) = \sum _{\begin{array}{c} E_1 \uplus E_2 = E \\ B_1 \uplus B_2 = B \\ C_1 \uplus C_2 = C \end{array}} f(E_1,B_1,C_1)g(E_2,B_2,C_2)I_{B_1,B_2}I_{C_1,C_2}(-1)^{|E_1|(|B_2|+|C_2|)}. \end{aligned}$$

We want to show that *Ham* is isomorphic (see Definition 10) with a product of all $H_D$ for $D \subseteq U$ (see Definition 9). In particular, diagram (14) commutes.

14

where $\tau _D: Ham \longrightarrow H_D$ is given as

$$\begin{aligned} (\tau _D f)(E,B,C) = I_{B,E}I_{C,E}\sum _{A \subseteq D} f(A,B\cup E,C\cup E). \end{aligned}$$

### Lemma 7

Transformation $\tau$ and its inverse can be performed in time $O^*(6^{tw})$.

### Corollary 1

Transformation $\tau$ is reversible.

### Lemma 8

Given $f, g \in H_D$ we can compute $f\oslash g$ in time $O^*(2^{\omega |D|}2^{|U\backslash D|})$.

### Lemma 9

Diagram (14) commutes, i.e., $\tau$ is a homomorphism of algebras.

Since $\tau$ is a reversible homomorphism of algebras of the same dimension $6^{tw}$ we obtain the following corollary.

### Corollary 2

Transformation $\tau$ is an isomorphism of algebras.

As for the Clifford algebras, we can switch the representation of the algebra to perform multiplication in the simpler one, and then revert the isomorphism to get the result. The most time consuming part of the algorithm is performing the $\oslash$ convolutions. Total number of operations modulo polynomial factor can be bounded with Lemma 8 by

15 $$\begin{aligned} \sum _{D \subseteq U} 2^{\omega |D|}2^{|U\backslash D|} = \sum _{k=0}^{tw} \left( {\begin{array}{c}tw\\ k\end{array}}\right) 2^{\omega k}2^{tw-k} = (2^{\omega } + 2)^{tw}. \end{aligned}$$

The rest of the appendix is devoted to proving Lemmas 7, 8 and 9.

### Proof of Lemma 7

For fixed sets *B*, *C* let $H = B \cap C,\, F = B \triangle C,\, B_1 = B \backslash C,\, C_1 = C \backslash B$. Observe that every allowed triple (*A*, *B*, *C*) must satisfy $A \cap F = C_1$. Therefore we can represent *Ham* as a union of sets

$$\begin{aligned} T_{B_1,C_1,H} = \Big \{(A_1 \cup C_1, B_1 \cup H, C_1 \cup H)\,\Big |\,A_1 \subseteq U\backslash (B_1 \cup C_1)\Big \} \end{aligned}$$

for all pairwise disjoint triples $B_1, C_1, H \subseteq U$. Functions over $T_{B_1,C_1,H}$ can be parameterized with only the $A_1$ argument. Consider following transformation over function space on $T_{B_1,C_1,H}$.

$$\begin{aligned} (\gamma _{B_1,C_1,H} f)(A_1) = \sum _{A_0 \subseteq A_1} f(A_0 \cup C_1, B_1 \cup H, C_1 \cup H) \end{aligned}$$

Transform $\gamma _{B_1,C_1,H}$ is just the Möbius transform, therefore it can be performed and inverted in time $O^*(2^{|U\backslash (B_1 \cup C_1)|})$ (see Theorem 3). Values of $\gamma f$ correspond directly to values of $\tau f$, that is, after computing values $(\gamma _{B_1,C_1,H} f)(A_1)$ for all allowed arguments we can read all values $(\tau _D f)(E,B,C)$ and vice versa. We check this by unrolling the formulas.

$$\begin{aligned} (\tau _D f)(E,B,C)&= I_{B,E}I_{C,E}\sum _{A \subseteq D} f(A,B\cup E,C\cup E) \\&= I_{B,E}I_{C,E}\sum _{A \subseteq D} f(A,B_1\cup H \cup E, C_1\cup H\cup E) \\&= I_{B,E}I_{C,E}\sum _{A_0 \subseteq D \backslash F} f(A_0 \cup C_1 ,B_1\cup H \cup E, C_1\cup H\cup E) \\&= I_{B,E}I_{C,E}(\gamma _{B_1,C_1,H\cup E} f)(D \backslash F) \\ \\ (\gamma _{B_1,C_1,H} f)(A_1)&= \sum _{A_0 \subseteq A_1} f(A_0 \cup C_1, B_1 \cup H, C_1 \cup H) \\&= \sum _{A_0 \subseteq A_1 \cup C_1} f(A_0, B_1 \cup H, C_1 \cup H) \\&= \sum _{A_0 \subseteq A_1 \cup C_1} f\big (A_0, B_2 \cup (H \backslash A_1), C_2 \cup (H \backslash A_1)\big ) \\&=(\tau _{A_1 \cup C_1} f)(E,B_2,C_2)I_{B_2,E}I_{C_2,E} \end{aligned}$$

where $E = H\backslash A_1,\, B_2 = B_1 \cup (H \cap A_1),\, C_2 = C_1 \cup (H \cap A_1)$ are valid arguments of $\tau _{A_1 \cup C_1}$.

To estimate the total number of operations consider all choices of *F*. The partition into $F = B_1 \uplus C_1$ can be done in $2^{|F|}$ ways, the set *H* can be chosen in $2^{|U\backslash F|}$ ways, and for such triple we have to perform the $\gamma _{B_1,C_1,H}$ transform (or its inverse) what involves $O^*(2^{|U\backslash F|})$ operations. Hence, the total running time (modulo polynomial factors) is

$$\begin{aligned} \sum _{F \subseteq U} 2^{|F|}4^{|U\backslash F|} = \sum _{k=0}^{tw} \left( {\begin{array}{c}tw\\ k\end{array}}\right) 2^{k}4^{tw-k} = 6^{tw}. \end{aligned}$$

$$\square $$

### Proof of Lemma 8

Applying the size-grouping (see Remark 1) allows us to neglect factor $(-1)^{|E_1|(|B_2|+|C_2|)}$ and replace condition $E_1 \uplus E_2 = E$ with $E_1 \cup E_2 = E$. Therefore it suffices to perform the $\odot$ convolution on $H_D$ (the same as in (13)).

$$\begin{aligned} (f \odot g)(E,B,C) = \sum _{\begin{array}{c} E_1 \cup E_2 = E \\ B_1 \uplus B_2 = B \\ C_1 \uplus C_2 = C \end{array}} f(E_1,B_1,C_1)g(E_2,B_2,C_2)I_{B_1,B_2}I_{C_1,C_2}. \end{aligned}$$

Let us denote

$$\begin{aligned} (\mu _E f)(B,C) = \sum _{F \subseteq E} f(F, B, C). \end{aligned}$$

Transform $\mu$ and its inverse can be computed using Möbius transform (see Theorem 3) in time $O^*(2^{|U\backslash D|})$ for all *E* and a fixed pair of sets *B*, *C*. We perform it for all $4^{|D|}$ such pairs.

It turns out that $\mu$ is an isomorphism between $(H_D, \odot )$ and a product of all algebras given by images of $\mu _E$ for $E \subseteq U\backslash D$ (see Definitions 9, 10) with multiplication given by NSC2, i.e., $(\mu _E f) \diamond _2 (\mu _E g) = \mu _E (f \odot g)$. We can again switch the representation of the algebra, multiply the elements on all coordinates given by *E*, and then revert the isomorphism. The computations below show that $\mu$ is a homomorphism of algebras and we know already that $\mu$ is reversible.

$$\begin{aligned} \big ((\mu _E f) \diamond _2 (\mu _E g)\big )(B, C)&= \sum _{\begin{array}{c} B_1 \uplus B_2 = B \\ C_1 \uplus C_2 = C \end{array}} (\mu _E f)(B_1,C_1)(\mu _E g)(B_2,C_2)I_{B_1,B_2}I_{C_1,C_2} \\&= \sum _{\begin{array}{c} E_1,E_2 \subseteq E \\ B_1 \uplus B_2 = B \\ C_1 \uplus C_2 = C \end{array}} f(E_1,B_1,C_1)g(E_2,B_2,C_2)I_{B_1,B_2}I_{C_1,C_2} \\&= \sum _{F \subseteq E} \sum _{\begin{array}{c} E_1 \cup E_2 = F \\ B_1 \uplus B_2 = B \\ C_1 \uplus C_2 = C \end{array}} f(E_1,B_1,C_1)g(E_2,B_2,C_2)I_{B_1,B_2}I_{C_1,C_2} \\&=\big (\mu _E (f \odot g)\big )(B, C)&\end{aligned}$$

To perform multiplication of $\mu (a)$ and $\mu (b)$, where $a,b \in H_D$, we have to perform NSC2 ($O^*(2^{\omega |D|})$ time complexity, see Theorem 6) for each $E \subseteq U\backslash D$, what results in desired running time. $\square$

### Proof of Lemma 9

We need to show that for each $B, C\subseteq D, D \cap E = \emptyset$ it is $(\tau _D (f \odot g))(E,B,C) = ((\tau _D f) \oslash (\tau _D g))(E,B,C)$. Let us start with unrolling the formula for $\tau _D (f \odot g)$. Keeping in mind that $B \cap E = C \cap E = \emptyset$ we can see that

16 $$\begin{aligned}&(\tau _D (f \odot g))(E,B,C) \nonumber \\&\quad =\sum _{\begin{array}{c} \quad A \subseteq D\quad \end{array}}(f \odot g)(A,B\cup E, C \cup E)I_{B,E}I_{C,E}\nonumber \\&\quad = \sum _{\begin{array}{c} A_1, A_2 \subseteq D \\ B_1 \uplus B_2 = B \\ E_1 \uplus E_2 = E \\ C_1 \uplus C_2 = C \\ F_1 \uplus F_2 = E \end{array}} \genfrac{}{}{0.0pt}{}{f(A_1,B_1 \cup E_1,C_1\cup F_1)g(A_2,B_2\cup E_2,C_2\cup F_1)}{I_{B_1 \cup E_1, B_2\cup E_2}I_{C_1\cup F_1, C_2\cup F_2}I_{B,E}I_{C,E}.} \end{aligned}$$

On the other hand, we have

17 $$\begin{aligned}&((\tau _D f) \oslash (\tau _D g))(E,B,C) \nonumber \\&\quad =\sum _{\begin{array}{c} E_1 \uplus E_2 = E \\ B_1 \uplus B_2 = B \\ C_1 \uplus C_2 = C \end{array}} (\tau _D f)(E_1,B_1,C_1)(\tau _D g)(E_2,B_2,C_2)I_{B_1,B_2}I_{C_1,C_2}(-1)^{|E_1|(|B_2|+|C_2|)}\nonumber \\&\quad = \sum _{\begin{array}{c} A_1, A_2 \subseteq D \\ E_1 \uplus E_2 = E \\ B_1 \uplus B_2 = B \\ C_1 \uplus C_2 = C \end{array}} \genfrac{}{}{0.0pt}{}{f(A_1,B_1\cup E_1,C_1\cup E_1)g(A_2,B_2\cup E_2,C_2\cup E_2)}{I_{B_1,B_2}I_{C_1,C_2}I_{B_1,E_1}I_{C_1,E_1}I_{B_2,E_2}I_{C_2,E_2}(-1)^{|E_1|(|B_2|+|C_2|)}.} \end{aligned}$$

We want to argue that all non-zero summands of (16) satisfy $$E_1 = F_1, E_2 = F_2$$. Indeed, let us assume $$v \in F_1 \backslash E_1$$. As $$v \in E$$ so $$v \not \in D \supseteq A, B, C$$ and $$\big ([v \in A_1], [v \in B_1 \cup E_1],$$$$[v \in C_1 \cup F_1]\big ) = (0, 0, 1)$$ which is not a valid triple what implies $$f(A_1,B_1 \cup E_1,C_1\cup F_1) = 0$$.

Assumption $v \in E_1 \backslash F_1$ leads to $\big ([v \in A_1], [v \in B_1 \cup E_1], [v \in C_1 \cup F_1]\big ) = (0, 1, 0)$ but $v \in E = E_1 \uplus E_2 = F_1 \uplus F_2$ so $\big ([v \in A_2], [v \in B_2 \cup E_2], [v \in C_2 \cup F_2]\big ) = (0, 0, 1)$ and $g(A_2,B_2\cup E_2,C_2\cup F_1) = 0$. The same arguments can be used if $v \in E_2 \triangle F_2$.

Now we just need to prove that for $E_1 = F_1, E_2 = F_2$ the *I* factors in (16) and (17) are equivalent. We apply Observation 2 to $I_{B_1 \cup E_1, B_2\cup E_2}I_{C_1\cup E_1, C_2\cup E_2}$. We can omit factor $I^2_{E_1,E_2} = 1$ as well as $I_{B_1,B_2}I_{C_1,C_2}$ appearing also in (17). What is left to prove is that

$$\begin{aligned}&I_{B_1,E_2}I_{E_1,B_2}I_{B,E} = I_{B_1,E_1}I_{B_2,E_2}(-1)^{|E_1||B_2|}, \\&I_{C_1,E_2}I_{E_1,C_2}I_{C,E} = I_{C_1,E_1}I_{C_2,E_2}(-1)^{|E_1||C_2|}. \end{aligned}$$

According to Observation 1 we can replace $I_{E_1,B_2}(-1)^{|E_1||B_2|}$ with $I_{B_2,E_1}$ what reduces the formula in the first row to Observation 2 for $B = B_1 \uplus B_2,\, E = E_1 \uplus E_2$. Applying analogous transformation to the second row finishes the proof. $\square$

## Footnotes

1 It can be found on the list of open problems from 2014 FPT school (http://fptschool.mimuw.edu.pl/opl.pdf) stated as *different convolution*.

2 Clifford algebras with $q=0$ appear also in geometric literature as *exterior algebras*.

3 As confirmed by the authors (private communication with Marek Cygan), the formula in [4] for the join node is missing the first argument to the $A_x$ function tracking the number of vertices of a Steiner tree, hence we present a corrected version of this formula.
